# Primary hepatic perivascular epithelioid cell tumors: imaging findings with histopathological correlation

**DOI:** 10.1186/s40644-019-0212-x

**Published:** 2019-06-06

**Authors:** Pei Nie, Jie Wu, Hexiang Wang, Ruizhi Zhou, Lingling Sun, Jingjing Chen, Guangjie Yang

**Affiliations:** 1 Department of Radiology, the Affiliated Hospital of Qingdao University, Qingdao, 266000 Shandong China; 2 Department of Pathology, the Affiliated Hospital of Qingdao University, Qingdao, 266000 Shandong China; 3 PET-CT Center, the Affiliated Hospital of Qingdao University, Qingdao, 266000 Shandong China

**Keywords:** Perivascular epithelioid cell tumors, Liver, CT, MRI, Ultrasound, Pathology

## Abstract

**Background:**

Hepatic PEComas are very rare. Few systematic reports are available characterizing the imaging and pathological features of hepatic PEComa. The aim of this study was to investigate the imaging findings of primary hepatic perivascular epithelioid cell tumors (PEComa) and its correlation with histopathological features.

**Methods:**

The CT, MRI and ultrasound images and pathological findings of 22 patients with primary hepatic PEComa were retrospectively reviewed.

**Results:**

More females (14/22) were affected with the mean age of 47.1 years. Most patients (17/22) were asymptomatic and the routine laboratory tests were normal. More tumors occurred in the right lobe (13/22) with a mean diameter of 76.7 mm. Surgery was performed in 21 patients, and biopsy was performed in 1 patient. Immunohistochemical studies showed the expression rate of HMB-45 and Melan A was 100% (22/22) and 86.4% (19/22) within the tumor cells. The pathology diagnoses were angiomyolipoma (*n* = 18), lymphangioleiomyoma (*n* = 2), clear-cell myomelanocytic tumor of falciform ligament/ligamentum teres (n = 1), and not otherwise specified (n = 1). Fifteen cases were classified as uncertain malignant potential (*n* = 13) or malignant (n = 2). CT, MRI and ultrasound features included well-defined margins (19/22), internal heterogeneity (20/22), arterial enhancement (20/22), dysmorphic vessels (17/22), fat (9/22), hemorrhage (3/22), necrosis (8/22), and calcification (2/22). The diagnostic accuracy was only 27.3% (6/22). No local recurrence or metastasis was found in the follow-up patients (12/22).

**Conclusions:**

On CT, MRI and ultrasound images, most hepatic PEComas are well-defined, heterogeneous, arterial enhanced masses with dysmorphic vessels, with or without fat, especially in middle-aged females. With the potential to be malignant, timely surgical resection and long-term follow-up may be helpful for improving the prognosis.

## Background

In 2013, the World Health Organization defined perivascular epithelioid cell tumors (PEComas) as mesenchymal tumors composed of distinctive perivascular epithelioid cells (PECs) that show a focal association with blood vessel walls and usually express melanocytic and smooth-muscle markers [1]. The PEComa family includes angiomyolipoma (AML), clear cell sugar tumor of lung (CCST), lymphangioleiomyoma /lymphangioleiomyomatosis (LAM), and a group of immunohistochemically similar tumors which includes primary extrapulmonary sugar tumor, clear-cell myomelanocytic tumor (CCMMT) of falciform ligament/ligamentum teres, abdominopelvic sarcoma of PECs and PEComa arising at a variety of soft tissue and visceral sites [1, 2]. PEComas show a wide anatomical distribution, but most arise in the uterus, retroperitoneum, abdominopelvic region and gastrointestinal tract [3]. Hepatic PEComas are very rare. Few systematic reports are available characterizing the imaging and pathological features of hepatic PEComa. The present study was undertaken to report the characteristics and correlation of imaging and histopathological findings in 22 cases to improve the understanding of this disease.

## Methods

### Patients

This retrospective study received approval from the institutional review board of our hospital and informed consent was not required. From September 2005 to October 2018, 22 patients with hepatic PEComa which were pathologically confirmed were enrolled. The patients’ demographics, clinical presentation, laboratory tests and tumor information were reviewed.

### Pathological examination

The pathologic diagnosis of PEComa was confirmed through surgery (*n* = 21) and image-guided percutaneous biopsy (*n* = 1). The tumor tissue was examined by hematoxylin-eosin (HE) staining and HMB-45 (human melanoma black monoclonal antibody), Melan A, Smooth muscle actin (SMA), S-100, Synaptophysin (Syn), Vimentin, Desmin, CK (creatine kinase), CD10, CD31, CD34, Hepatocyte, GPC3, Arginase-1, Cg A (chromogranin A), AFP (alpha feto protein), EMA (epithelial membrane antigen) and Ki-67 immunohistochemical staining. All the diagnoses were reviewed and confirmed by a single pathologist with expertise in liver pathology.

### CT, MRI and ultrasound imaging

This study spanned a period of 13 years. During this period, the images were acquired on a variety of CT, MRI and ultrasound modalities using a variety of technical parameters. The CT scans included non-enhanced and 3-phase dynamic contrast-enhanced examinations. The following sequences were acquired for MRI studies: in-phase and out-of-phase T1WI, fat-suppressed T2WI, DWI images for non-enhanced MRI; and pre- and post-IV contrast images for patients who received IV contrast. Pretreatment imaging of the primary tumor was available in all the patients including non-enhanced CT in 13 patients, contrast-enhanced CT in 18 patients, MRI in 9 patients, and ultrasound in 13 patients. One patient underwent gadolinium contrast-enhanced MRI 1 month after percutaneous artery embolization, and the CT scans before and after treatment were also obtained. Of the 9 patients who underwent MRI, 7 were performed both non-enhanced and contrast enhanced MRI scanning, 3 were administered the hepatocyte-specific agent gadoxetic acid (Primovist, Bayer Healthcare, Berlin, Germany) and 4 were administered gadolinium contrast material. The arterial phase, portal venous phase and delayed phase scans of dynamic CT and MRI were obtained at 25–30 s, 60–70 s, and 90–120 s respectively after the initiation of contrast administration; for the 3 patients who underwent Primovist-enhanced MRI, the hepatobiliary phase scans were obtained at 20 min after contrast injection. Ultrasonography was performed on 13 patients.

### Image analysis

All images were evaluated by two experienced abdominal radiologists who were blinded to the histopathological results. For any disagreement between the two observers, consensus agreement was achieved. The following imaging features of each tumor were assessed: number, location, size (the maximum diameter), margin, shape, internal consistency (homogeneous vs. heterogeneous), and presence of fat, necrosis, hemorrhage, calcification, and dysmorphic vessels. Dysmorphic vessels were regarded as prominent or enlarged vessels or arteriovenous shunting. The attenuation, signal intensity and echotexture relative to the background liver was recorded as low, isointense, or high. When available, the enhancement patterns of arterial, portal venous and delayed phases relative to the background liver were analyzed.

## Results

### Patients

There were more women in this group (14/22). Patient’s ages ranged from 23 to 76 years (mean age: 47.1 years). One patient presented with intermittent abdominal pain, 2 patients presented with severe pain, and 2 patients presented discomfort of upper abdomen. The other 17 patients were incidentally found to have a mass on physical examination. None of these patients was associated with tuberous sclerosis complex (TSC). Hepatitis B surface antigen, and hepatitis C virus antigen were negative, and the serum AFP, CEA, and CA19–9 levels were all within the normal range. The follow-up rate was 54.6% (12/22). Enlargement was not identified for the biopsy-confirmed patient (*n* = 1) and local recurrence or metastasis were not found during the follow-up of 2 to 98 months in 11 patients.

### Pathological findings

The cut surface of the tumors was grey-yellow or grey-red, soft, some had hemorrhage and/or necrosis, and most had clear boundaries between the tumor edge and normal tissue. Histological examination demonstrated that the tumor was highly cellular, with large round or polygonal epithelioid cells and abundant eosinophilic cytoplasm. Immunohistochemical studies showed a strong and diffuse expression of HMB-45 and Melan A within the tumor cells. The expression rate of HMB-45 and Melan A was 100% (22/22) and 86.4% (19/22), respectively. The pathology diagnoses showed that 18 cases (18/22) were AML, 2 cases (2/22) were LAM, 1 case (1/22) was CCMMT, and 1 case was not otherwise specified (NOS). Seven cases (7/22) were classified as benign, 13 cases (13/22) were classified as uncertain malignant potential, and 2 cases (2/22) were classified as malignant.

### Imaging findings

Eighteen patients had CT, 9 patients had MRI, 13 patients had ultrasound, 7 patients had both CT and MRI, and 5 patients had CT, MRI as well as ultrasound for review. All the patients had single lesion in the liver. Thirteen tumors (13/22) were located in the right lobe of the liver, 7 tumors in the left lobe and 2 tumors in the caudate lobe. The tumors size ranged from 1.4 to 23.6 cm in diameter (mean diameter: 76.7 mm). Twenty-one tumors were round or oval, and 1 tumor was slightly lobulated. Most tumors (19/22) had well-defined margins.

Internal heterogeneity was present in most cases (20/22). Fat was present in 40.9% (9/22) of tumors: 44.4% (8/18) of AML and 1 case of NOS. Fat showed similar attenuation to fat tissue on CT or fat signal intensity decreased on out-of-phase images compared with the in-phase images. Four cases demonstrated more than 50% region of the tumor was occupied by the fat, the other 5 cases showed scattered, foliated fat within the tumors. Necrosis was present in 36.4% of tumors (8/22) giving a non-enhanced cystic appearance. Hemorrhage with high-density on non-enhanced CT or high signal intensity on fat-suppressed T1WI images was identified in 3 tumors (3/22), and calcification was identified in 2 cases (2/22) in the form of punctate foci on CT. Dysmorphic vessels were identified in 77.3% (17/22) tumors by contrast enhanced CT or MRI examinations. All the tumors were found to be in livers without a background of cirrhosis or hepatitis. The portal vein or bile duct system were not invaded, and there was no abnormal lymph node or distant metastasis.

On non-enhanced CT of 13 patients, all the tumors showed low attenuation compared to the background liver. On non-enhanced MRI, 6 out of 7 tumors were low in signal intensity on T1WI, and high in signal intensity on fat-suppressed T2WI and DWI images, one tumor with large amount of fat showed low signal intensity on fat-suppressed T2WI and DWI images. Of the 13 patients with ultrasound examinations, most tumors were seen as well-circumscribed heteroechoic masses, and the internal blood flow was variable on Doppler ultrasound.

Twenty-one patients had contrast enhanced CT and/or MRI examinations. The enhancement patterns of the tumors were variable. Arterial enhancement was observed in 20 cases (20/22). Enhancement in the arterial phase with fast washout was identified in 9 cases (9/22); enhancement in the arterial phase with slow washout was observed in 7 cases (7/22); enhancement in the arterial phase with persistent enhancement in the late phases was observed in 4 cases (4/22); and unspecified heterogeneous enhancement pattern was noted in 2 cases (2/22). Of the 3 patients with hepatocyte-specific agent enhanced MRI, the hepatobiliary phase showed marked hypointensity of the tumor relative to the liver parenchyma. The features on CT, MRI and ultrasound are shown in Table 1.

**Table 1:** Features on CT, MRI and ultrasound

Imaging features	Total, *n* = 22 (%)
Right lobe	13 (59.1)
Round and oval	21 (95.5)
Well-defined margin	19 (86.4)
Heterogeneous	20 (90.9)
Fat	9 (40.9)
Necrosis	8 (36.4)
Hemorrhage	3 (13.6)
Calcification	2 (9.1)
Dysmorphic vessels	17 (77.3)
Features on non-enhanced CT	Total, n = 13 (%)
Low attenuation	13 (100)
Features on non-enhanced MRI	Total, n = 7 (%)
T1w low SI	6 (85.7)
T1w high SI	1 (14.3)
Fat-suppressed T2w low SI	1 (14.3)
Fat-suppressed T2w high SI	6 (85.7)
Features on enhanced CT and/or MRI	Total, n = 21 (%)
Arterial enhancement	20 (95.2)
Arterial enhancement with fast washout	9 (42.9)
Arterial enhancement with slow washout	7 (33.3)
Arterial enhancement with persistent enhancement in the late phases	4 (19.0)
Unspecified heterogeneous enhancement	1 (4.8)

**Fig. 1:**
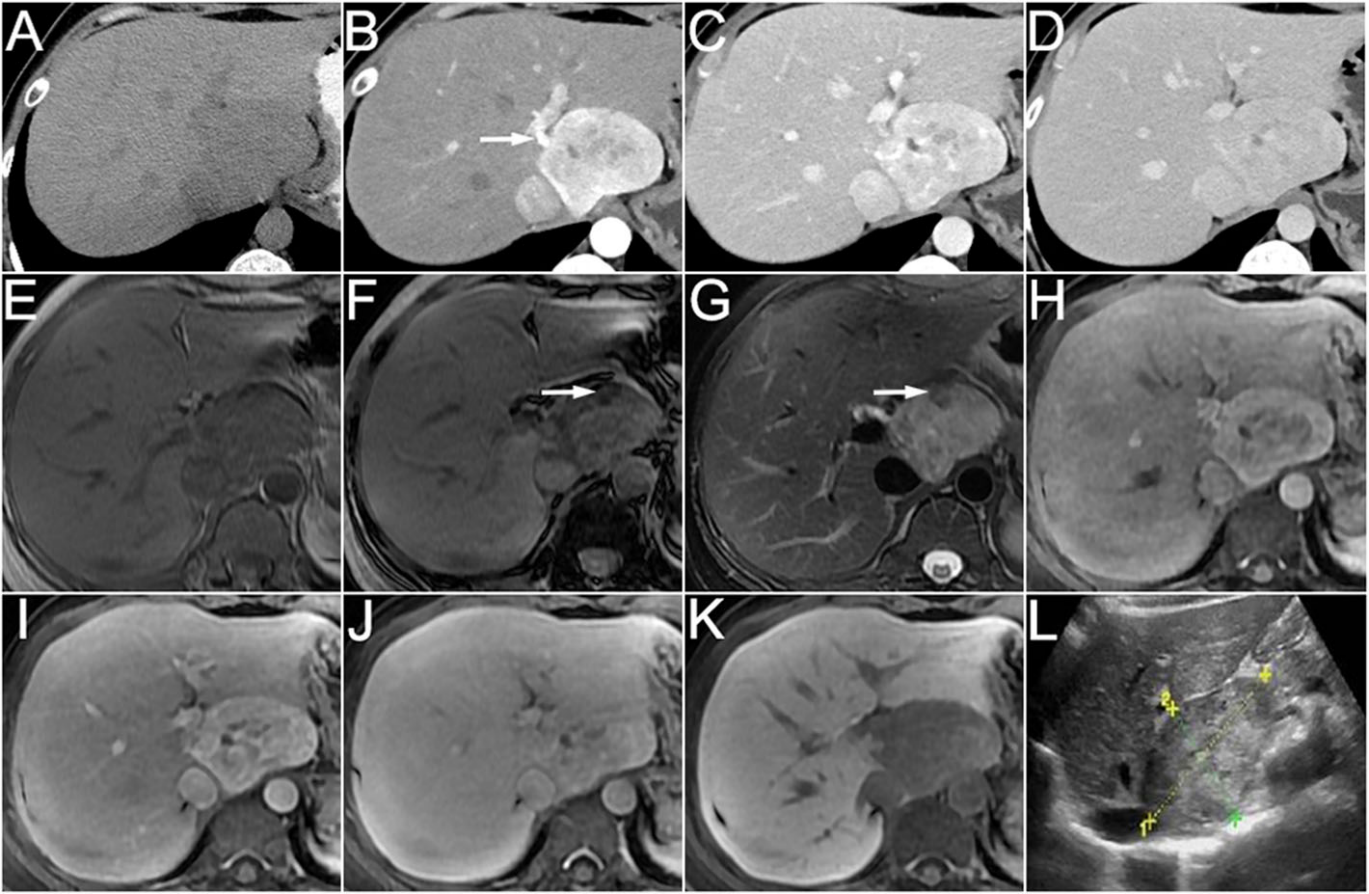
A 35-year woman with hepatic angiomyolipoma. The non-enhanced CT (**a**) showed a round well-circumscribed homogeneous hypoattenuated mass in the caudate hepatic lobe. On the arterial phase (**b**), the tumor demonstrated significantly heterogeneous enhanced surrounded by a dysmorphic vessel (arrow). On the portal venous (**c**) and delayed (**d**) phases, the enhancement decreased, but still showed higher density compared to the surrounding liver. The MRI (**e**, T1WI in-phase; **f**, T1WI out-of-phase; **g**, fat-suppressed T2WI) images showed hypointense on T1WI and hyperintense on T2WI. Note the fat showed signal intensity decreased on out-of-phase image as compared with in-phase image and low signal intensity on fat-suppressed T2WI (arrow). On hepatocyte-specific agent enhanced MRI (**h**, arterial phase; **i**, portal venous phase; **j**, delayed phase; **k**, hepatobiliary phase), the tumor showed the same enhancement on arterial, portal venous and delayed phase images as CT and marked hypointensity of the tumor relative to the liver parenchyma on the hepatobiliary phase image. Ultrasound (**l**) showed a well-defined heteroechoic mass in the liver

**Fig. 2:**
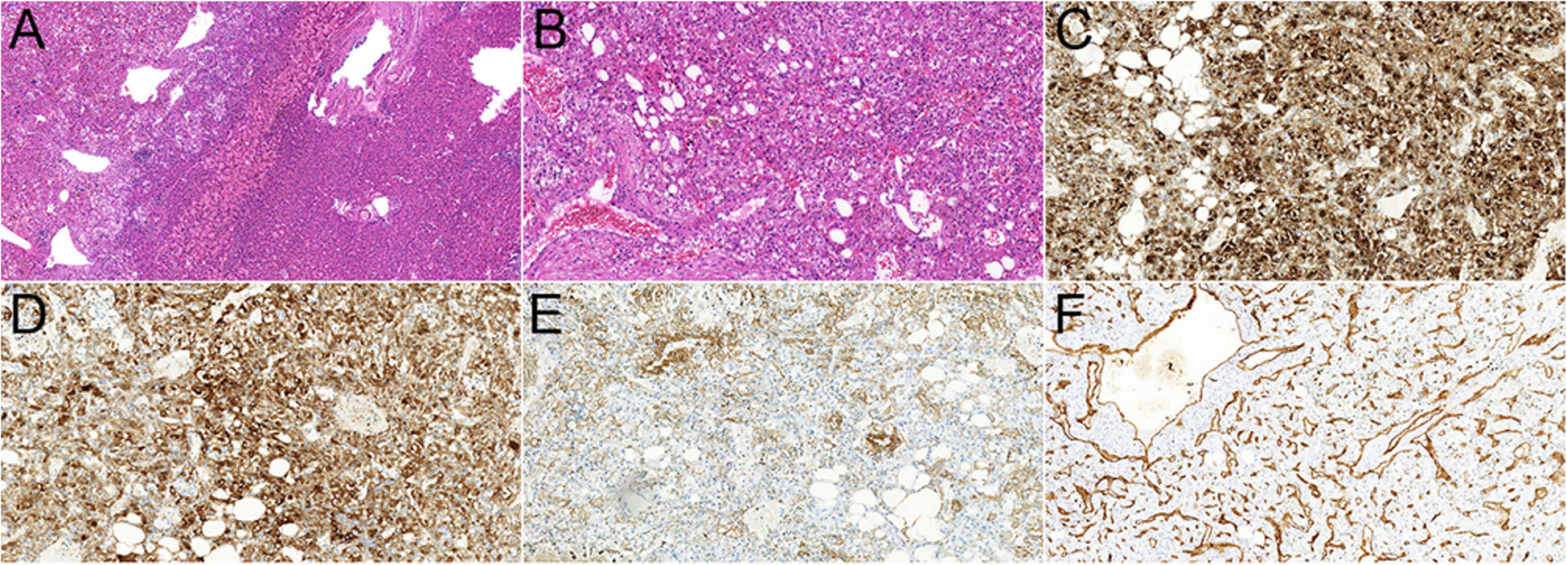
Histological examination of the 35-year woman with hepatic angiomyolipoma. H&E staining images (**a**, magnification × 100; **b** magnification × 200) demonstrated that the tumor mainly consisted of three components: the dilated thick-walled vessels; the spindle smooth muscle cells and epitheloid cells in a perivascular location; and some mature lipid tissues. Immunohistochemistry demonstrated the cells were positive for HMB-45 (**c**, magnification × 200), Melan A (**d**, magnification × 200), SMA (**e**, magnification × 200), and CD34 (**f**, magnification × 200)

**Fig. 3:**
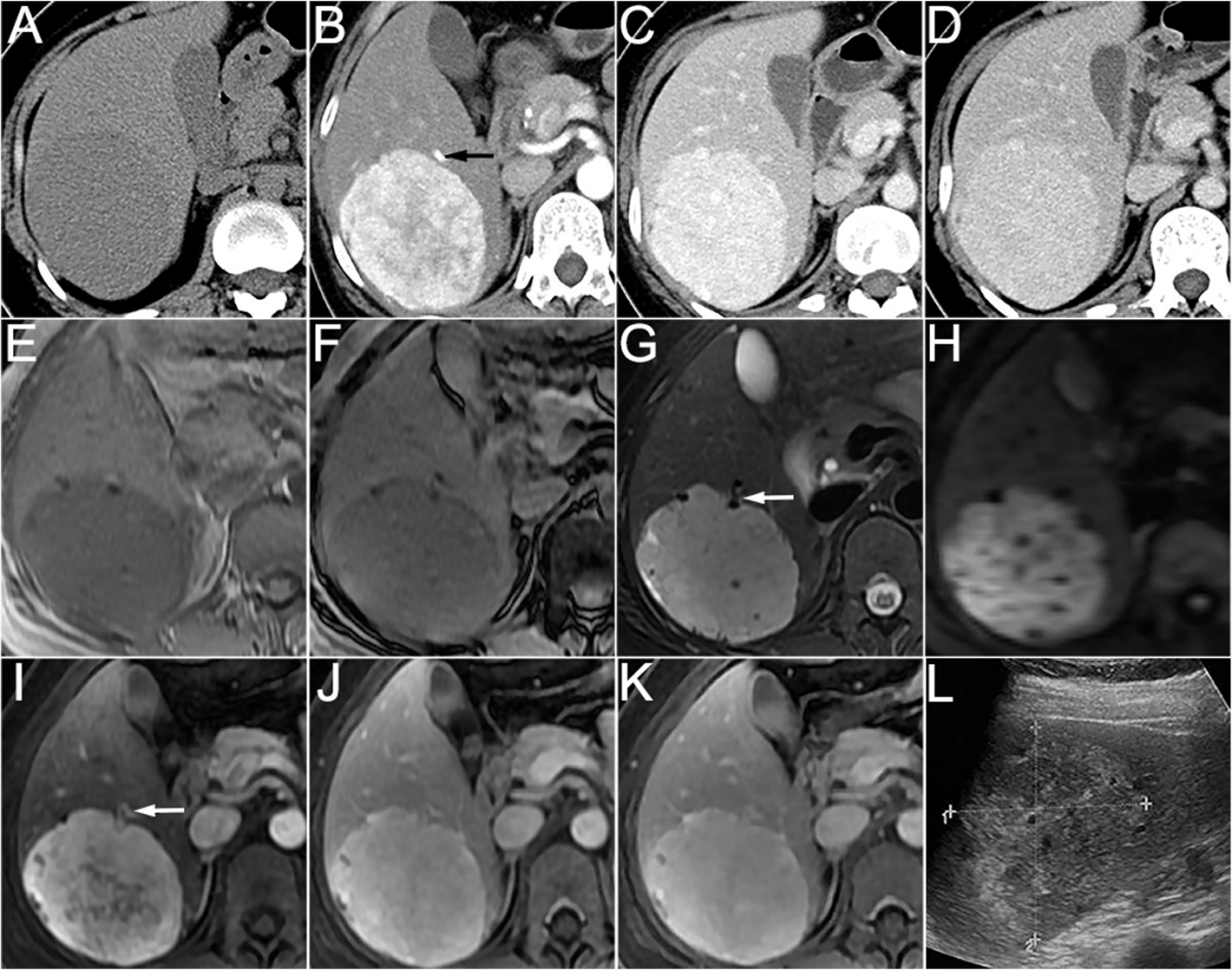
A 39-year woman with hepatic lymphangioleiomyoma. The non-enhanced CT scan (**a**) revealed a round well-defined homogeneous hypoattenuated mass in the right hepatic lobe. On the arterial phase (**b**), the mass demonstrated significantly heterogeneous enhanced surrounded by a dysmorphic vessel (arrow). On the portal venous (**c**) and delayed (**d**) phases, the mass became homogeneous, but still showed higher attenuation compared to the background liver. The MRI scan (**e**, T1WI in-phase; **f**, T1WI out-of-phase; **g**, fat-suppressed T2WI; **h**, DWI) showed hypointense on T1WI, hyperintense on T2WI and high signal intensity on DWI. The enhancement pattern on dynamic contrast-enhanced MRI (**i**, arterial phase; **j**, portal venous phase; **k**, delayed phase) was corresponded with CT. Note the vessel around the tumor on T2WI and arterial phase images (arrow). Ultrasound (**l**) showed a round, well-defined hyperechoic mass with a cribriform appearance in the right lobe of liver

**Fig. 4:**
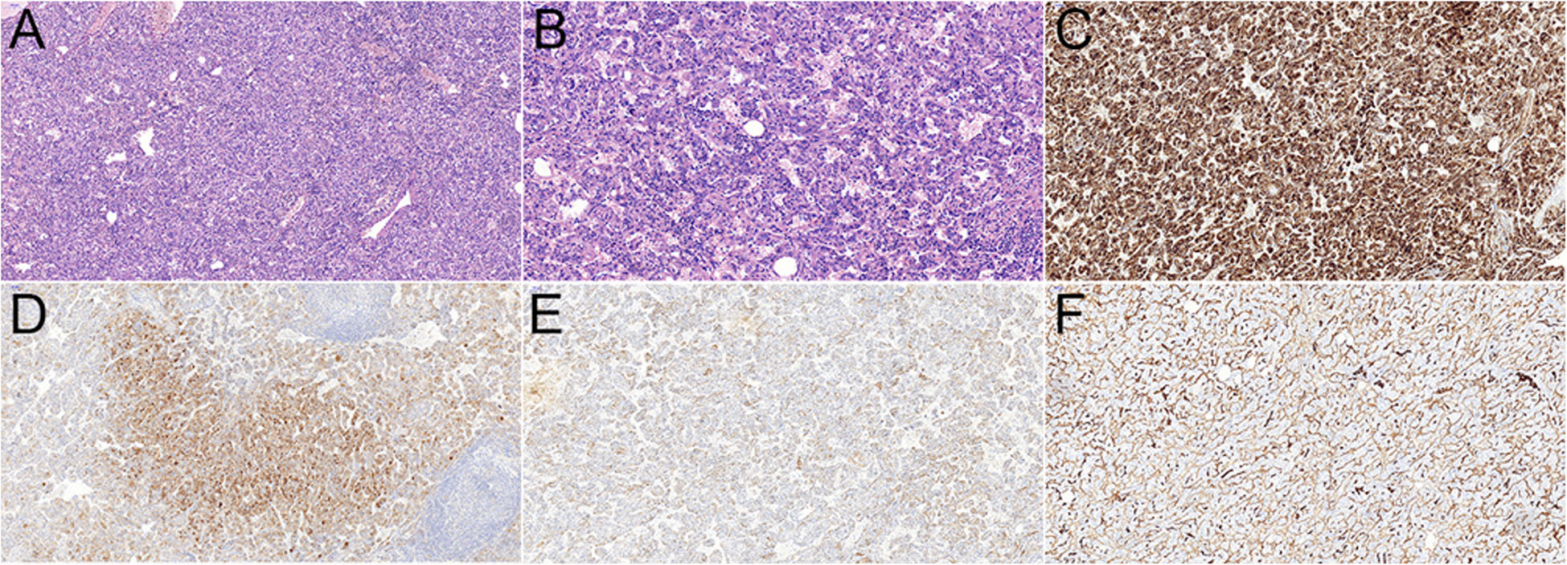
Histological examination of the 39-year woman with hepatic lymphangioleiomyoma. H&E staining images (**a**, magnification × 100; **b** magnification × 200) demonstrated that the tumor mainly consisted of the spindle smooth muscle cells and epitheloid cells. Immunohistochemistry demonstrated the cells were positive for HMB-45 (**c**, magnification × 100), Melan A (**d**, magnification × 100), SMA (**e**, magnification × 100) and CD34 (**f**, magnification × 100)

**Fig. 5:**
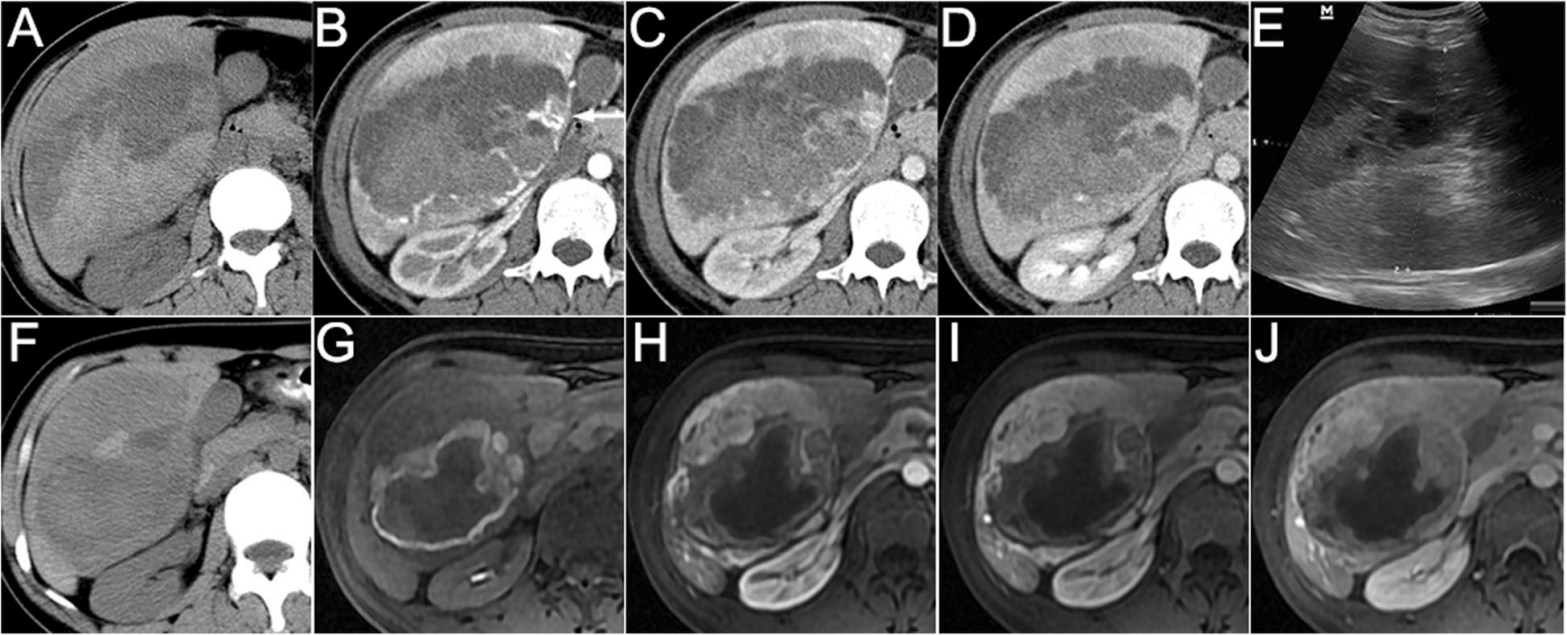
A 29-year woman with clear-cell myomelanocytic tumor of falciform ligament/ligamentum teres. This patient presented acute pain of upper abdomen and was hospitalized in the emergency department. The non-enhanced CT (**a**) showed a large heterogeneous mass in the right hepatic lobe with high and low attenuation. Contrast-enhanced CT images (**b**, arterial phase; **c**, portal venous phase; **d**, delayed phase) demonstrated heterogeneous enhancement with necrosis and hemorrhage within the mass. Note the mass was surrounded by dysmorphic vessels (arrow) on arterial phase image. Ultrasound (**e**) showed an ill-defined, large hyperechoic mass with hypoechoic region within the lesion in the right lobe of liver. The patient was performed percutaneous artery embolization. One month later, she underwent CT and contrast-enhanced MRI to reevaluate the mass. Necrosis, hemorrhage and heterogeneous enhancement were also identified on non-enhanced CT (**f**) and contrast-enhanced MRI (**g**, mask T1WI; **h**, arterial phase; **i**, portal venous phase; **j**, delayed phase) images. Note the tumor diameter was decreased from 130 mm to 97 mm

**Fig. 6:**
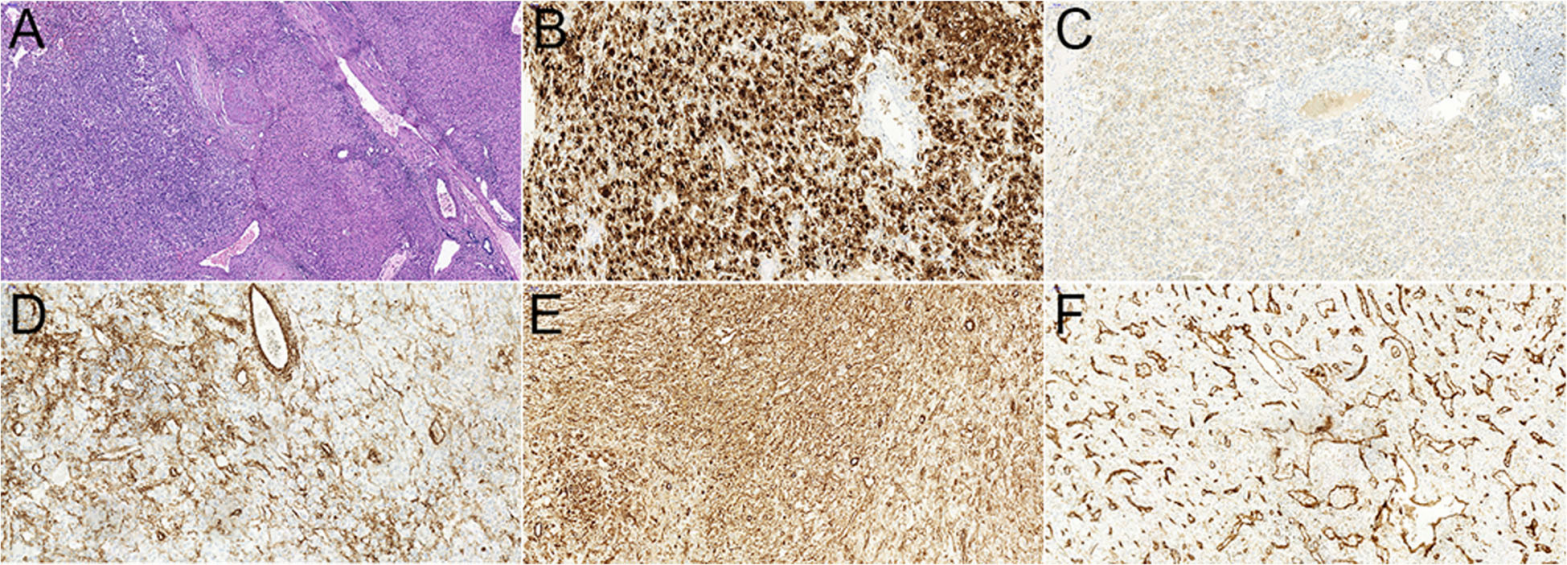
Histological examination of the 29-year woman with clear-cell myomelanocytic tumor of falciform ligament/ligamentum teres. H&E staining images (**a**, magnification × 100) demonstrated the dilated thick-walled vessels and perivascular spindled cells and epitheloid cells with clear to lightly eosinophilic cytoplasm. Immunohistochemistry demonstrated the cells were positive for HMB-45 (**b**, magnification × 200), Melan A (**c**, magnification × 200), SMA (**d**, magnification × 200), Vimentin (**e**, magnification × 200) and CD34 (**f**, magnification × 200)

Using pathology as the reference standard, the radiological diagnostic accuracy before surgery or biopsy was only 27.3% (6/22). All the 6 cases showed fat on CT/MRI images and were diagnosed as AML confirmed by pathology. Other 16 cases including AML without fat (*n* = 12), LAM (*n* = 2), CCMMT (n = 1) and NOS (n = 1) were misdiagnosed as hepatocellular carcinoma (HCC, *n* = 6), hepatocellular adenoma (*n* = 4), focal nodular hyperplasia (FNH, n = 1), hepatocellular adenoma or FNH (*n* = 3), and hemangioma (n = 2). Images and pathological findings of 3 cases of hepatic PEComa (AML, 1; LAM, 1; CCMMT, 1) are shown in Figs. 1, 2, 3, 4, 5 and 6.

## Discussion

PEComa is a rare mesenchymal tumor composed of histologically and immunohistochemically distinctive PECs. In 1992, Bonetti firstly proposed the concept of PECs to describe an “unusual atypical cell type” which typically has a perivascular distribution, with dual melanocytic and myoid differentiation [4]. The term PEComa was first introduced by Zamboni 4 years later [5, 6]. PEComas are predominant in the uterus, and primary hepatic PEComas appear to be less common than other PEComas. Until today, only a few cases of hepatic PEComa have been described worldwide [7–10].

Previous studies report that PEComas are commonly seen in women during the fifth decade of life [1, 5]. Abdominal pain, nausea, indigestion, and loss of appetite have been reported, but most patients are asymptomatic or have nonspecific gastrointestinal symptoms [1]. Our 22 cases arose in 14 women and 8 men, with a mean patient age of 47.1 years (range from 23 to 76 years). Seventeen tumors are found incidentally. Three patients presented with abdominal pain, and 2 patients presented discomfort. Two patient presented severe abdominal pain and were found the tumors were very large and ruptured. The size of tumors in our group was relatively larger with a mean diameter of 76.7 mm. The tumors often occurred in the right lobe (13/22), and showed no background of hepatitis or cirrhosis. Routine laboratory tests of PEComa patients are usually noncontributory.

The histogenesis and pathogenesis of PECs are remains uncertain. One hypothesis is that it may originate from undifferentiated neural crest cells with the capability of expressing the phenotype of melanocytes and smooth muscles. A smooth muscle origin or a pericytic origin have been considered to be other possibilities [1]. Genetic studies have found that PEComa may occur as a sporadic disease or as a component of TSC (including TSC1 and TSC2), which suggests the oncogenesis of PEComas as a TSC-associated neoplasm [11].

PEComa can be readily diagnosed by its characteristic pathological findings. Histologically, PEComa is characterized by the proliferation of cells with epithelioid appearance closely related to dilated vascular channels. Typically, the cells in an immediate perivascular location are most epithelioid, whereas spindled cells resembling smooth muscle are seen further away from vessels. Lipid may be found accumulated within PECs. PECs have clear to lightly eosinophilic cytoplasm rather than the dense eosinophilia of true smooth muscle cells. The PEC is characterized by coexpression of melanocytic markers, such as HMB-45, Melan-A, tyrosinase, and micropthalmia transcription factor; and muscle markers, such as SMA, pan-muscle actin, muscle myosin, and calponin [11]. In our study, HMB45 and Melan A were the most sensitive melanocytic marker of PEComa, similar to the published results.

The “PEComa family” now comprises AML, CCST, LAM, CCMMT and other NOS. AML was the most common pathological type, the other pathological types were relatively rare [12, 13]. Although the majority of the reported PEComas seem to behave in a benign fashion, a malignant course of these tumors, with local recurrence and distant metastasis, has also been reported [4, 14]. The criteria for malignancy of hepatic PEComas have not yet been fully established. Folpe et al. [11] proposed a categorization of PEComas into benign, uncertain malignant potential (UMP), and malignant neoplasm. The worrisome features include large than 5 cm in size, infiltrative, high nuclear grade and cellularity, mitotic activity more than 1/50 high power fields (HPF), coagulative necrosis or vascular invasion. PEComas with none of the above worrisome features are benign, while PEComas with one of these features are of UMP, and PEComas with two or more features are defined as malignant. These criteria were observed in the present cases, and 13 cases (13/22) were classified as UMP, and 2 cases (2/22) were classified as malignant.

PEComas exhibit a wide spectrum of imaging findings [14, 15]. Hepatic PEComas can be of any echogenicity with blood flow in or surrounding the lesion. For the cases with fat, especially in those lesions with fat occupying more than 50% of the tumor, the possible diagnosis of AML can be easily made. The diagnosis is challenging in the tumors with no or little fat. On non-enhanced CT and MRI, most of the tumors show low attenuation, low signal intensity on T1WI, and high signal intensity on fat-suppressed T2WI and DWI images. These findings are non-specific in most liver tumors. Previous reports have suggested hypervascularity and arteriovenous connections as a feature of PEComa [15]. Our study had the similar results, as arterial enhancement was observed in most cases (20/22), and 77.3% of the cases showed dysmorphic vessels in or surrounding the tumors. However, the enhancement patterns of the tumors were variable including enhancement in the arterial phase with fast washout (*n* = 9), enhancement in the arterial phase with slow washout (*n* = 7), enhancement in the arterial phase with persistent enhancement in the late phases (*n* = 4) and unspecified heterogeneous enhancement pattern (*n* = 2). This radiological variation may be explained with the proportion of different components within the tumors, such as adipose tissue (very variable, from less than 10% to over 90%), blood vessels, and smooth muscle cells [3].

Making a pre-operative diagnosis radiologically seems to be very difficult. In the present study, the proportions of pathological types of hepatic PEComa were AML (18/22), LAM (2/22), CCMMT (1/22) and NOS (1/22). The diagnostic accuracy before surgery was only 27.3% (6/22). Six cases of AML with fat on CT/MRI images were diagnosed accurately. The other 16 patients were misdiagnosed as HCC (*n* = 6), hepatocellular adenoma (*n* = 4), FNH (*n* = 1), hepatocellular adenoma or FNH (*n* = 3), or hemangioma (*n* = 2) based on the imaging findings. The following points may offer some help in differential diagnosis. Most HCC are found to be in livers with a background of cirrhosis or hepatitis in China and are associated with relatively high AFP levels. Most cases of FNH are smaller than 5 cm and typical FNH has a central stellate scar showing hyperintensity on T2WI and delayed enhancement on late-phase images. Lack of the stellate scar and hypointensity on the hepatobiliary phase images should be recognized as distinguishing clues from FNH. On T2-weighted images, hemangioma is usually markedly hyperintense owing to their vascular nature. Most hepatic PEComas showed relatively lower signal intensity on T2WI than typical hemangioma, and may not show the progressive centripetal fill-in enhancement. Hepatocellular adenoma is typically sharply marginated with a pseudocapsule. It usually demonstrates arterial fast enhancement with slow washout on delayed phase images. Larger adenomas display heterogeneous attenuation/intensity because of necrosis, fat, hemorrhage and calcification. These imaging features of adenomas overlap with those of some hepatic PEComas making the differential diagnosis rather difficult.

Surgical resection is the most commonly used strategy because of the uncertain behavior of PEComa. Recently it has been demonstrated that PEComas are generated by the proliferation of PECs with mutations leading to loss of TSC gene activity resulting in overexpression of the kinase mammalian target of rapamycin (mTOR) [16, 17]. Inhibitors of mTOR may therefore provide new treatment options. Systemic neoadjuvant treatment with sirolimus has been used to obtain tumor shrinkage and facilitate surgical removal [18, 19]. Malignant PEComas treated with transarterial embolization, radiofrequency ablation and stereotactic body radiation therapy were also reported [20, 21]. One of our patients was performed percutaneous artery embolization before surgery, and the tumor was found decreased in size one month after the treatment. Although most PEComas are benign, there are certain cases that imply invasive growth, with distant metastasis or recurrences. Although no local recurrence or metastasis was found in the follow-up patients (12/22). Long-term periodic follow-up is required due to the uncertain clinical behavior of these tumors [22].

Certain limitations of our study deserve consideration. First, a relatively small group of patients were included, future studies with larger patient populations are required. Second, the CT, MRI and ultrasound were performed on different modalities using a variety of technical parameters. Lack of standardization of imaging parameters may influence image analysis. Third, the follow-up rate was 54.6% in this study, other tumors without follow-up were not evaluated.

## Conclusions

In summary, primary hepatic PEComas are rare tumors. Although imaging features are nonspecific and the diagnosis is based on histopathology, PEComas should be considered in the differential diagnosis of hepatic masses with well-defined margins, arterial enhancement and dysmorphic vessels, with or without fat. Most of the tumors occurred in asymptomatic middle-aged females. PEComas can display characteristics of both benign and malignant tumors. Timely surgical resection and long-term follow-up may be helpful for improving the prognosis.

## Abbreviations

AML: Angiomyolipoma
CCMMT: Clear-cell myomelanocytic tumor
CCST: Clear cell sugar tumor of lung
FNH: Focal nodular hyperplasia
HE: Hematoxylin-eosin
HMB: Human melanoma black monoclonal antibody
LAM: Lymphangioleiomyoma/lymphangioleiomyomatosis
mTOR: Mammalian target of rapamycin
nos: Not otherwise specified
PEC: Perivascular epithelioid cell
PEComa: Perivascular epithelioid cell tumor
SMA: Smooth muscle actin
TSC: Tuberous sclerosis complex
UMP: Uncertain malignant potential
